# Lipid metabolism as a target for cancer drug resistance: progress and prospects

**DOI:** 10.3389/fphar.2023.1274335

**Published:** 2023-09-28

**Authors:** Zi’an Wang, Yueqin Wang, Zeyun Li, Wenhua Xue, Shousen Hu, Xiangzhen Kong

**Affiliations:** ^1^ Department of Pharmacy, The First Affiliated Hospital of Zhengzhou University, Zhengzhou, China; ^2^ Henan Key Laboratory of Precision Clinical Pharmacy, Zhengzhou University, Zhengzhou, China; ^3^ Department of Otolaryngology Head and Neck Surgery, The First Affiliated Hospital of Zhengzhou University, Zhengzhou, China

**Keywords:** drug resistance, lipid metabolism, combination drug, inhibitor, chemotherapy resistance, targeted therapy

## Abstract

Cancer is the world’s leading cause of human death today, and the treatment process of cancer is highly complex. Chemotherapy and targeted therapy are commonly used in cancer treatment, and the emergence of drug resistance is a significant problem in cancer treatment. Therefore, the mechanism of drug resistance during cancer treatment has become a hot issue in current research. A series of studies have found that lipid metabolism is closely related to cancer drug resistance. This paper details the changes of lipid metabolism in drug resistance and how lipid metabolism affects drug resistance. More importantly, most studies have reported that combination therapy may lead to changes in lipid-related metabolic pathways, which may reverse the development of cancer drug resistance and enhance or rescue the sensitivity to therapeutic drugs. This paper summarizes the progress of drug design targeting lipid metabolism in improving drug resistance, and providing new ideas and strategies for future tumor treatment. Therefore, this paper reviews the issues of combining medications with lipid metabolism and drug resistance.

## 1 Introduction

Lipid metabolism is a fundamental and intricate biochemical process within the human body. The primary biochemical process of lipid metabolism involves phospholipid and cholesterol metabolism, which are regulated by various factors such as insulin, glucagon, dietary nutrition, and enzymatic activities. Through this complex process, lipids are converted into essential components that are necessary for a wide range of biochemical reactions within the body (Liu et al., 2022).

The significance of lipid metabolism extends to the development, of cancer (Li et al., 2023), as cancer cells heavily rely on it to acquire the energy, biofilm constituents, and signaling molecules essential for their proliferation, survival, invasion, and metastasis (Bian et al., 2021). Unlike normal cells, cancer cells undergo a series of modifications in lipid metabolism, which can have a profound impact on the increased efflux of anti-tumor drugs and the modulation of apoptotic signaling pathways. These modifications consequently influence the development of tumor drug resistance.

Chemotherapy and targeted therapies currently serve as the primary treatment for cancer (Tse et al., 2021). These interventions hold the potential to improve the overall survival and prognosis of cancer patients (Chen et al., 2023). However, the emergence of drug resistance presents a substantial clinical challenge that needs to be overcome. This challenge applies not only to the conventional chemotherapeutic agents commonly used in the initial stages but also to the targeted agents that are currently undergoing active development and investigation (Zeng et al., 2019).

The development of drug resistance in cancer is influenced by various factors, and its mechanisms can be broadly categorized into mutations in drug targets and metabolism, inhibition of apoptosis, activation of intracellular survival signaling pathways, enhanced DNA repair, immune evasion by cancer stem cells (CSCs), and metabolic abnormalities, and so on (Ramos and Bentires-Alj, 2015; Pan et al., 2016). While previous studies on cancer drug resistance mainly focused on genetic mutations and external factors, cancer metabolism have been as a new research focal point in recent years (Zhao et al., 2013; Ma and Zong, 2020). An increasing number of studies suggest that the development of resistance to chemotherapy and targeted therapies is closely linked to metabolic alterations, including lipid metabolism, which can affect the sensitivity of cancer cells to drugs.

Studies have indicated that the utilization of combination therapies targeting multiple pathways may effectively delay the development of therapeutic resistance (Fitzgerald et al., 2006). Combination drug therapy represents an emerging and more potent approach to treatment administration. By employing different mechanisms, drug combinations can collectively work towards achieving therapeutic objectives. Additional drugs can sequentially intervene in disease-related signaling pathways, either through the same or different routes, thereby producing synergistic effects (Yin et al., 2014). Moreover, synergistic drug combinations have the potential to reduce the required dosage of individual drugs within the mixture, consequently diminishing drug toxicity and mitigating the risk of drug resistance.

To further understand enhance our understanding of lipid metabolism and its connection to drug resistance and to investigate the relevance of drug combination, this review will focusconcentrate on five aspects: alterationkey areas: the modification of lipid metabolism in cancer cells, the association between phospholipid metabolism and drug resistance, the impact of cholesterol metabolism andon drug resistance, the involvement of microRNA andin lipid metabolism and drug resistance, and the significance of drug combination and lipid metabolism andin relation to lipid metabolism and drug resistance. Through exploring these aspects, we aim to gain deeper insights into the intricate relationship between lipid metabolism and the development of drug resistance.

## 2 Alteration of lipid metabolism in cancer cells

Extensive researches have been dedicated to exploring the intricate relationship between lipid metabolism and cancer development. Lipid metabolism plays a crucial role in providing signaling molecules (Boroughs and DeBerardinis, 2015), essential substrates for phospholipid synthesis (Zaidi et al., 2013), and metabolic fuels for mitochondrial oxidation (An et al., 2022). By modulating these pathways, lipid metabolism exerts control over the growth and proliferation of cancer cells. Cancer cells exhibit distinct patterns of nutrient uptake and utilization compared to normal cells, leading to a series of modifications in lipid metabolism. These metabolic alterations drive the growth and proliferation of cancer cells and even contribute to the development of resistance against conventional anticancer therapies.

Most normal cells primarily generate energy through mitochondrial oxidative phosphorylation, a process that involves the transfer of electrons from NADH or FADH_2_ to O_2_ via a series of mitochondrial electron carriers (Viña et al., 2009). However, in contrast to normal cells, many cancer cells rely on high-rate glycolytic and lactic acid fermentation pathways, a phenomenon known as the Warburg effect (Koppenol et al., 2011). Although aerobic glycolysis is less efficient in producing ATP compared to oxidative phosphorylation, it generates other metabolites that support tumor growth (Ashrafian, 2006). Lipid supply is crucial for the proliferation and survival of various cancer cells (Liang and Dai, 2022), and previous studies have demonstrated that cancer cells predominantly acquire lipids through the *de novo* fatty acid synthesis pathway (Baron et al., 2004). Activation of this pathway is believed to be necessary for carcinogenesis (Zhou et al., 2007).

**FIGURE 1:**
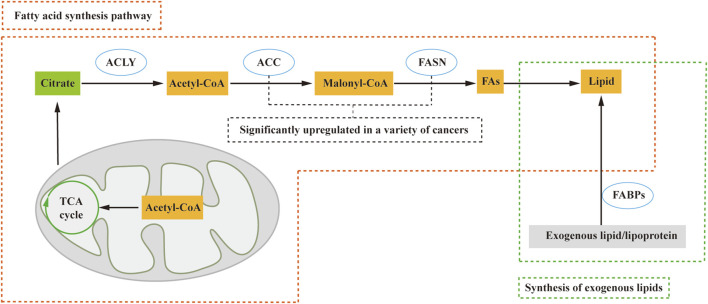
Lipid sources include the *de novo* fatty acid synthesis pathway and exogenous lipid uptake. In this process, citric acid from the tricarboxylic acid cycle (TCA cycle) crosses the mitochondrial membrane and enters the cytosol. Subsequently, ATP citrate lyase (ACLY) releases Acetyl-CoA, which plays a crucial role in the fatty acid synthesis pathway. Acetyl-CoA is then catalyzed by acetyl-CoA carboxylase (ACC) to form malonyl-CoA (Malony-CoA), and fatty acid synthase (FASN) further catalyzes the synthesis of fatty acids and, consequently, lipids. Additionally, fatty acid-binding proteins (FABPs) facilitate the conversion of exogenous lipids and lipoproteins into lipids. ACLY, ATP citrate lyase; ACC, acetyl-CoA carboxylase; FA, fatty acid; FABPs, fatty acid-binding proteins; FASN, fatty acid synthase; TCA cycle, tricarboxylic acid cycle.

Due to limited oxygen and extracellular nutrients, most cancer cells synthesize fatty acids *de novo.* The process of fatty acid synthesis occurs in the cytosol, with acetyl CoA serving as the starting material. However, acetyl CoA, although present in the mitochondria (Guertin and Wellen, 2023), cannot directly traverse the mitochondrial membrane, necessitating a transport mechanism to enter the cytosol. In contrast, citric acid produced in the tricarboxylic acid cycle (TCA) can cross the mitochondrial membrane and enter the cytosol. Within the cytosol, acetyl CoA is released from citrate by citrate lyase (ACLY) and participates in fatty acid synthesis. Acetyl CoA is subsequently converted to malonyl CoA by acetyl-CoA carboxylase (ACC), followed by the action of fatty acid synthase (FASN) in synthesizing lipids (Tanosaki et al., 2020) (Figure 1). Key regulators such as FASN and ACC are significantly upregulated in various human cancers, such as cervical cancer and breast cancer (Menendez and Lupu, 2017; Du et al., 2022).

Nevertheless, certain cell types, including proliferating fibroblasts, HeLa, and H460 cells (Yao et al., 2016), exhibit a preference for direct uptake of lipids from the extracellular environment rather than *de novo* synthesis. These cells convert exogenous lipids and lipoproteins into the necessary lipids for cell growth and proliferation, facilitated by fatty acid-binding proteins (FABPs) (Ntambi, 2022). According to these studies, lipid acquisition depends on the type of cell and microenvironment, whether through *de novo* fatty acid synthesis or alternative pathways, and contributes to tumorigenesis.

## 3 Phospholipid metabolism and drug resistance

Lipids containing phosphate are called phospholipids. Phospholipids can divide, which contain phosphate, play a crucial role in various biological processes. They can be categorized into two main groups: those made up of glycerol are called phosphoglycerides, composed of glycerol, and those made up of neurosphingosine are called sphingolipids, composed of sphingosine.

Glycerophospholipids are the most abundant phospholipids found in the body, serving multiple essential functions. They not only form the structural basis of biological membranes but also contribute to bile composition, act as surface-active substances, and play a role in protein recognition and cell membrane signaling. Glycerophospholipids can be classified into various categories based on their substitution groups, with some of the most important ones being phosphatidylcholine (PC) formed by choline and phosphatidic acid, phosphatidylethanolamine (PE) formed by ethanolamine and phosphatidic acid, phosphatidylserine (PS) formed by serine and phosphatidic acid, phosphatidylglycerol (PG) formed by glycerol and phosphatidic acid, and phosphatidylinositol (PI) formed by inositol and phosphatidic acid (Ntambi, 2022). The synthesis of glycerophospholipids occurs through three stages: raw material sourcing, activation, and glycerophospholipid generation. This process takes place in the endoplasmic reticulum of the cytoplasm, undergoes processing by the Golgi apparatus, and is ultimately utilized by tissue biofilms or secreted as lipoproteins. Glycerophospholipids can be synthesized in various body tissues, excluding mature erythrocytes. In living organisms, certain phospholipases can hydrolyze glycerophospholipids, and their degradation mainly involves hydrolysis catalyzed by different phospholipases in the body. During glycerophospholipid metabolism, several bioactive lipid molecules are generated, including inositol triphosphate, glycerol diacyl, arachidonic acid, phosphatidic acid, and lysophosphatidic acid. These lipid molecules, in turn, regulate diverse intracellular signaling pathways (Oude Weernink et al., 2007).

Sphingolipids, distinguished by the absence of glycerol and the presence of sphingomyelin, encompass sphingomyelin and glycosphingolipids. They are synthesized in various tissues throughout the body, with particularly high activity in brain tissues where they constitute a major component of neural tissue membranes. The synthesis of sphingolipids occurs within the endoplasmic reticulum. The breakdown of sphingolipids takes place through the hydrolysis of sphingolipids into choline phosphate and ceramide, catalyzed by phospholipase (Duan M. et al., 2022). Sphingomyelin, an important structural component of cell membranes, also serves as a precursor for various metabolites, including ceramide, ceramide-1-phosphate, sphingosine, sphingosine-1-phosphate, and glycosyl ceramide. These metabolites play crucial roles as bioactive lipid molecules involved in apoptosis and signaling pathways related to drug resistance.

### 3.1 Phospholipid metabolism and chemotherapy resistance

**FIGURE 2:**
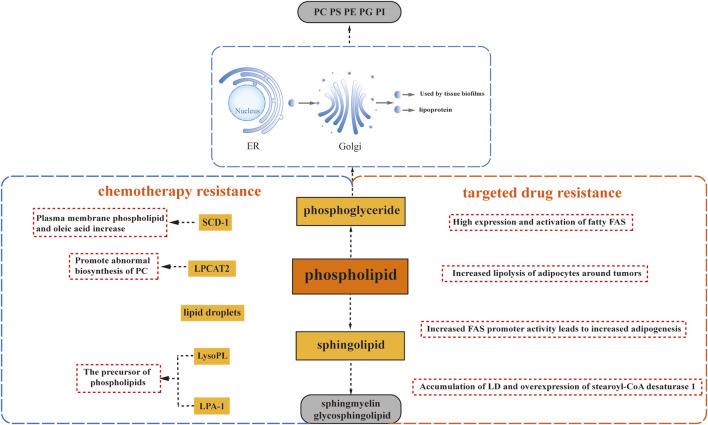
Phospholipid Metabolism and Drug Resistance. Phospholipid metabolism plays a crucial role in drug resistance, involving glycerophospholipids and sphingomyelins. Glycerophospholipids encompass phosphatidylcholine (PC), phosphatidylethanolamine (PE), phosphatidylserine (PS), phosphatidylglycerol (PG), and phosphatidylinositol (PI), each characterized by different substituents. Sphlngolipids comprise sphlngmyelins and glycosphingolipids. Several factors contribute to drug resistance: the abnormal biosynthesis of phosphatidylcholine driven by lysophosphatidylcholine acyltransferase 2 (LPCAT2), upregulation of Stearoyl-CoA desaturase 1 (SCD1) leading to lipid droplet accumulation, and the induction of drug resistance by precursors of phospholipids, namely, lysophospholipid (LysoPL) and lysophosphatidic acid receptor 1 (LPA-1).

The fatty acid composition of phospholipids (PL) plays a crucial role in distinguishing between sensitive and resistant cells. Recent studies have highlighted the impact of acyltransferases on the fatty acid composition of PL, which can influence cancer chemosensitivity. For instance, lysophosphatidylcholine acyltransferase 2 (LPCAT2), an enzyme associated with lipid droplets, has been found to promote abnormal biosynthesis of phosphatidylcholine, leading to resistance to oxaliplatin and 5-fluorouracil in colorectal cancer. The underlying mechanism involves an enhanced anti-apoptotic response to endoplasmic reticulum stressors and improved resistance to immunogenic cell death induced by chemotherapy (Cotte et al., 2018). These findings suggest that LPCAT2 activity can modify the lipid composition of the endoplasmic reticulum and plasma membrane, thereby reducing sensitivity to endoplasmic reticulum stress and impairing recognition by the host immune system (Figure 2).

Additionally, progesterone has been found to upregulate Stearoyl-CoA desaturase 1 (SCD1), resulting in an enrichment of oleic acid in plasma membrane phospholipids. This enrichment has been correlated with docetaxel resistance, as it increases the mobility of the plasma membrane and alters its connection to the cytoskeleton. Consequently, cells become better adapted to drugs that target the cytoskeleton, such as docetaxel (Schlaepfer et al., 2012) (Figure 2). Moreover, studies have reported a link between enhanced lipid droplet formation and drug resistance. In progesterone-dependent breast cancer, increased intracellular accumulation of lipid droplets has been associated with docetaxel resistance since hydrophobic cytotoxic drugs, including docetaxel, are readily sequestered within lipid droplets (Schlaepfer et al., 2012). Similarly, a significant increase in neutral lipids within lipid droplets and the accumulation of free cholesterol in lysosomes have been observed in a variant of T-47D breast cancer cells resistant to the lipid-soluble drug tamoxifen (Hultsch et al., 2018). The presence of lipid droplets has also been identified in MCF7R cells with acquired resistance to doxorubicin (Morjani et al., 2001) (Figure 2).

Lysophospholipids (LysoPL), the direct precursors of phospholipids (PL), also play a role in mediating drug resistance. Interestingly, their mechanism of action is not solely dependent on the plasma membrane. A recent study demonstrated that LysoPL containing long saturated fatty acyl chains can induce drug resistance. This protective effect of LysoPL enables tumor cells to withstand DNA-damaging agents like cisplatin, representing a lipid-specific and drug-specific protective mechanism (Kramer et al., 2015) (Figure 2).

Similarly, lysophosphate-1 (LPA-1), a precursor shared by most phospholipids, has been implicated in reducing the effectiveness of adriamycin in inhibiting the viability of triple-negative MDA-MB-231 cells with paclitaxel (Samadi et al., 2009) (Figure 2). Furthermore, it has been observed that LPA-1 upregulates several multidrug efflux transport proteins, including MRP1, MRP2, MRP3, and BCRP, along with various antioxidant enzymes (Venkatraman et al., 2015) (Figure 2). This implies that LPA-1 activates at least two mechanisms that promote resistance to chemotherapy.

### 3.2 Phospholipid metabolism and targeted therapy resistance

Alterations in phospholipid-related metabolism are closely associated with the development of resistance to targeted drugs, mirroring the pattern seen with many chemotherapeutic agents. In a comprehensive study conducted by Geneste *et al.*, it was revealed through both *in vitro* and *in vivo* validation that increased adipocyte lipolysis surrounding tumors contributes to the resistance of breast tumor cells to lapatinib-mediated cytotoxicity (Geneste et al., 2020) (Figure 2). Moreover, elevated expression and activation of adipogenic fatty acid synthase (FAS) were observed in breast, colon, and prostate cancers, leading to enhanced lipid droplet (LD) accumulation and synthesis of triacylglycerol (TAG) (Pandey et al., 2012; Wu et al., 2020).

Further investigations focusing on breast cancer cells have demonstrated that the development of trastuzumab resistance is associated with heightened adipogenesis due to increased FAS promoter activity (Menendez et al., 2004) (Figure 2). Similarly, the accumulation of LD and overexpression of stearoyl-CoA desaturase 1 (SCD1) were observed in non-small cell lung cancer (NSCLC) cells resistant to EGFR-tyrosine kinase inhibitors (TKIs) (Figure 2). Interestingly, the extent of LD accumulation resulting from upregulated adipogenesis was greater in EGFR/TKI-resistant cells with aberrantly activated EGFR signaling pathways than in cells harboring sensitive EGFR mutations. This observation aligns with the role of *de novo* adipogenesis driven by the upregulation of the receptor tyrosine kinase signaling pathway, particularly the maintenance of sterol regulatory element-binding protein (SREBP) activity (Butler et al., 2020).

## 4 Cholesterol metabolism and drug resistance

### 4.1 Cholesterol and its synthesis and metabolism

Cholesterol is a vital component of animal cell membranes, serving not only as a structural element but also as a precursor for the synthesis of bile acids, vitamin D, and steroid hormones (Griffiths and Wang, 2022). Its physiological significance in the human body is diverse and essential (Rezen et al., 2011).

**FIGURE 3:**
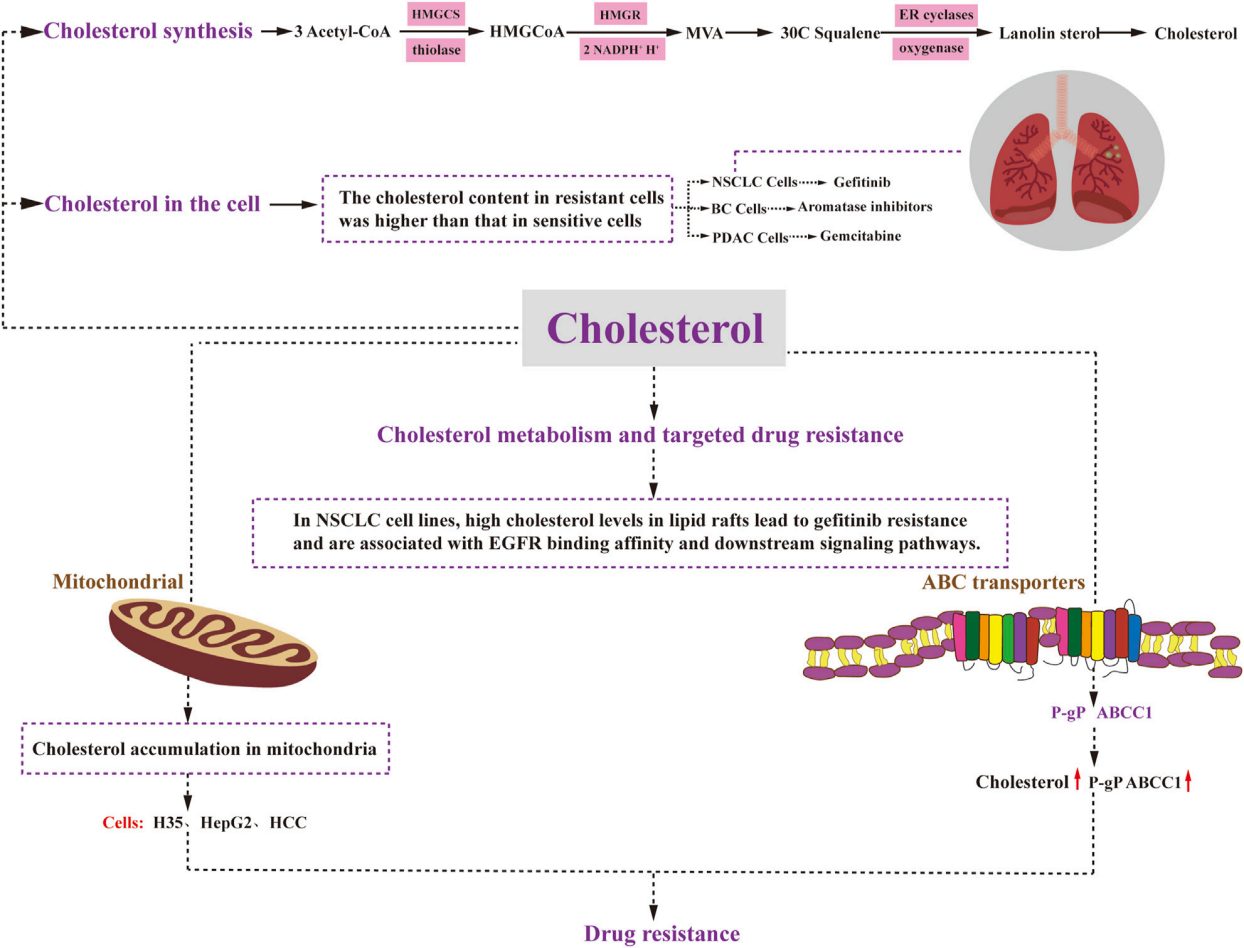
Cholesterol metabolism and drug resistance. Cholesterol synthesis involves three stages: HMGCoA generation, MVA generation, and cholesterol generation. Cholesterol plays a regulatory role in cancer cell resistance through various mechanisms. Elevated levels of mitochondrial cholesterol can contribute to resistance against apoptosis. Changes in the composition of cell membrane cholesterol can affect the function of ABC transporters, including P-glycoprotein and ABCC1. Moreover, alterations in cell membrane cholesterol content may impact the permeability of therapeutic agents and drug uptake. Ultimately, drug resistance in cancer cells affects critical cellular processes such as survival, proliferation, differentiation, and apoptosis. HMGCoA:3-Hydroxy-3-methylglutaryl coenzyme A; HMGCS: 3-Hydroxy-3-methylglutaryl coenzyme A synthetase; MVA: mevalonic acid; HMGR: HMGCoA reductase; ER cyclases: endoplasmic reticulum cyclases.

Cholesterol is synthesized in nearly all tissues of the body, with the liver being the primary site of synthesis, occurring predominantly in the cytosol and the endoplasmic reticulum. The process of cholesterol synthesis can be summarized into three stages (Figure 3). The first stage involves the production of 3-hydroxy-3-methylglutaryl CoA (HMG-CoA): Within the cytosol, three molecules of acetyl CoA undergo catalysis by Thiolase and HMG-CoA synthase (HMGCS) to form HMG-CoA. This process is similar to the production of ketone bodies, although it occurs in a different intracellular location. The second stage encompasses the generation of mevalonic acid (MVA): HMG-CoA is converted by HMG-CoA reductase (HMGR), consuming two molecules of NADPH^+^ and H^+^ to produce mevalonic acid (MVA). This step is irreversible. The third stage involves the production of cholesterol: MVA is initially phosphorylated, decarboxylated, and dehydroxylated, ultimately leading to the synthesis of 30C squalene. This squalene is then catalyzed by endoplasmic reticulum cyclase and hydrogenase to generate lanolin sterols, which further undergo a series of multi-step reactions, including redox reactions, to ultimately yield cholesterol (Ačimovič and Rozman, 2013).

### 4.2 Cholesterol homeostasis and drug resistance

The association between cholesterol homeostasis and drug resistance has been extensively investigated (Duan Y. et al., 2022). Studies using data from the Cancer Genome Atlas have shown a correlation between cholesterol synthesis, decreased patient survival, and cancer progression (Weinstein et al., 2013). Understanding the role of cholesterol in drug resistance is crucial for overcoming challenges in cancer treatment (Wu et al., 2015). To elucidate the mechanisms underlying drug resistance, it is important to examine the involvement of cholesterol in this process.

Preclinical studies have provided compelling evidence linking cholesterol metabolism to drug resistance in various types of cancer, including prostate, lung, pancreatic, and breast cancers (Guillaumond et al., 2015; Nguyen et al., 2015; Zhan et al., 2019; El-Kenawi et al., 2021). Notably, in aggressive prostate cancer (CRPC), researchers have discovered that macrophage-derived cholesterol influences drug resistance during treatment by affecting its transportation and metabolism-related effects. This finding serves as a valuable model for understanding the mutation landscape of CRPC, supported by experimental data (El-Kenawi et al., 2021).

In non-small cell lung cancer, gefitinib-resistant cells have been found to exhibit significantly higher cholesterol levels compared to their gefitinib-sensitive counterparts (Zhan et al., 2019) (Figure 3). Similarly, in breast cancer, increased endogenous cholesterol biosynthesis in aromatase inhibitor-resistant cells leads to the activation of estrogen receptor-α, which subsequently diminishes the effectiveness of statins and impedes cell invasion (Nguyen et al., 2015) (Figure 3). In the case of pancreatic ductal adenocarcinoma (PDAC), disruption of low-density lipoprotein receptor (LDLR) internalization leads to alterations in free versus esterified cholesterol levels. This effect becomes more pronounced *in vivo* when gemcitabine (GEM) is administered (Guillaumond et al., 2015) (Figure 3). GEM-resistant PDAC cells exhibit elevated levels of cholesteryl ester (CE) compared to their sensitive counterparts (Li et al., 2018). Therefore, targeting LDLR or acyl coenzyme A cholesterol acyltransferase (ACAT) to limit CE accumulation holds promise for enhancing the efficacy of GEM against PDAC.

### 4.3 Mitochondrial cholesterol levels and drug resistance

Elevated levels of cholesterol in mitochondria have been demonstrated to contribute to resistance against apoptotic signaling, leading to chemotherapy resistance in cancer (Montero et al., 2008). Considering the crucial role of mitochondria in apoptosis regulation and chemotherapy response, several studies have highlighted the association between cholesterol accumulation and mitochondria-targeted chemoresistance in cancer cells. For instance, both rat H35 and human HepG2 and HCC cells exhibit resistance to anticancer agents that target mitochondria and induce the opening of mitochondrial permeability transition pores through various mechanisms (Le Bras et al., 2006) (Figure 3). Subsequent investigations have revealed that the resistance to chemotherapeutic agents in H35 and HepG2 cells can be reversed by treatment with lovastatin, an inhibitor of HMG-CoA reductase (HMGCR) that inhibits cholesterol resynthesis and prevents cholesterol accumulation in the mitochondrial membrane (Smith and Land, 2012). Moreover, a significant increase in mitochondrial cholesterol content has been observed in liver cancer tissues compared to normal tissues (Kramer et al., 2015). In this context, inhibition of the mevalonate pathway has been shown to reduce mitochondrial cholesterol content and enhance the hepatocyte response to the mitochondria-targeting drug doxorubicin, thereby sensitizing the cancer cells to chemotherapy (Montero et al., 2008). Additionally, mitochondria in hepatocellular carcinoma cells demonstrate resistance to mitochondrial membrane permeabilization and various other stimuli, with elevated cholesterol levels observed in all cases. Previous research has demonstrated that knockdown of the mitochondrial cholesterol transport polypeptide, steroid acute regulatory protein, which is upregulated in hepatocellular carcinoma cells, leads to reduced cholesterol synthesis in mitochondria and increased sensitivity of cells to chemotherapy (Domínguez-Pérez et al., 2019).

### 4.4 ABC transporter protein cholesterol levels and drug resistance

The significance of ABC transporter proteins in the development of drug resistance in various cancers has been extensively studied for several decades. Numerous studies have demonstrated that altering the cholesterol composition of cell membranes also affects the activity of these ABC transporter proteins Recent research has indicated that P-gP substrates may preferentially accumulate in cholesterol-rich regions of the membrane, thus increasing P-gP transport activity (Subramanian et al., 2016). Consequently, elevated cholesterol levels can promote P-gP activity and mediate drug resistance. Consistent with this observation, in colon cancer cells, the quantity and transport activity of P-gP decrease as cholesterol synthesis is inhibited. Another ABC transporter protein, ABCC1, also appears to be regulated by cholesterol. Its function has been linked to its localization in cholesterol-rich membrane microstructure domains. When membrane cholesterol levels drop below 40%, ABCC1 partially relocates to the high-density fraction, resulting in reduced functionality (Marbeuf-Gueye et al., 2007). In summary, the localization and function of ABCC1 in cell membranes are regulated by cholesterol.

### 4.5 Cholesterol metabolism and drug resistance to targeted therapy

Patients with advanced non-small cell lung cancer (NSCLC) who are treated with epidermal growth factor receptor tyrosine kinase inhibitors (EGFR-TKIs) experience significant clinical benefits. However, they inevitably develop acquired resistance. Research has demonstrated that high cholesterol levels in lipid rafts contribute to gefitinib resistance in NSCLC cell lines and are associated with altered EGFR binding affinity and downstream signaling pathways. This groundbreaking study highlights that elevated cholesterol levels in lipid rafts play a pivotal role in inducing gefitinib resistance in NSCLC cells by impacting EGFR phosphorylation, downstream signaling pathways, and EGFR-TKI affinity (Irwin et al., 2011) (Figure 3). Moreover, combination therapy with lovastatin has shown a synergistic inhibitory effect on gefitinib-resistant cells, making the combination of lovastatin and gefitinib a promising treatment strategy for patients with gefitinib resistance (Chen et al., 2018).

An increasing body of evidence highlights the crucial role of cholesterol in cancer development. Researchers have focused on investigating the impact of cholesterol on the acquisition of drug resistance in cancer. Elevated cholesterol levels and alterations in protein expression related to cholesterol metabolism have been observed in different types of drug-resistant cancer cells. Collectively, dysregulated cholesterol metabolism emerges as a fundamental factor contributing to the development of drug resistance in multiple cancer types.

## 5 MicroRNA-mediated lipid metabolism may affect drug resistance

MicroRNAs (miRNAs) are short, non-coding RNA molecules composed of approximately 22 nucleotides. They are encoded by endogenous genes and play a crucial role in the post-transcriptional regulation of gene expression in both plants and animals. MiRNAs have been widely implicated in various biological processes and are closely associated with the regulation of gene expression. In the context of lipid metabolism homeostasis, previous studies have revealed a close relationship between miRNAs and lipid metabolism. Specifically, several miRNAs, such as miR-33, miR-128–1, miR-144, and miR-148a, have been identified to target and suppress the expression of ABCA1 and ABCG1 transporter proteins. These findings have been demonstrated in cultured cells and further validated through *in vivo* experiments (Gerin et al., 2010; Horie et al., 2010; Goedeke et al., 2015; Wagschal et al., 2015). Since ABC transporter proteins have been implicated in the development of drug resistance, it is important to investigate whether the inhibitory effect of miRNAs on these transporter proteins is correlated with the emergence of drug resistance. Furthermore, miR-33a and miR-33b have been found to inhibit the synthesis of fatty acid oxidase, which leads to a reduction in intracellular lipid renewal (Dávalos et al., 2011; Gerin et al., 2010). These observations highlight the multifaceted role of miRNAs in lipid metabolism and suggest their potential involvement in modulating cellular responses to lipid-related therapies and drug resistance. Further research is needed to unravel the intricate mechanisms underlying the regulatory effects of miRNAs on lipid metabolism and drug resistance, providing valuable insights for the development of therapeutic strategies targeting these processes.

The liver, being the primary site of lipid metabolism in the body, plays a pivotal role in maintaining lipid homeostasis. Among the miRNAs involved in this process, miR-122 is particularly abundant in the liver (Chang et al., 2004). It exerts regulatory control over various genes associated with cholesterol and fatty acid synthesis (Krützfeldt et al., 2005; Esau et al., 2006), thereby influencing lipid metabolism. Another key player in regulating lipid metabolism is miR-27b, which acts as a central regulatory hub (Vickers et al., 2013), Its paralog, miR-27a, has also been identified as a regulator of lipid metabolism in the liver (Zhang et al., 2017). These miRNAs contribute to the fine-tuning of lipid synthesis and metabolism, ensuring the proper balance of lipids in hepatic cells. Furthermore, miR-223 has been found to inhibit cholesterol biosynthesis and reduce cholesterol levels. This miRNA adds to the repertoire of miRNAs involved in the regulation of lipid metabolism, highlighting their potential impact on cellular lipid profiles. Collectively, these miRNAs orchestrate a series of intricate associations with the development of drug resistance in cancer, potentially through their regulatory roles in lipid metabolism. Understanding the interplay between miRNAs, lipid metabolism, and drug resistance in cancer holds promise for the identification of novel therapeutic targets and strategies. Further investigation into these mechanisms will shed light on the underlying complexities of cancer biology and may pave the way for innovative approaches to combat drug resistance.

## 6 Combination of lipid metabolic pathways to alleviate tumor drug resistance

Combination therapy has emerged as a highly effective therapeutic approach employed in the treatment of various diseases (Han et al., 2017). Extensive research has demonstrated that combining multiple drugs offers several advantages over single-drug treatment, including enhanced efficacy, reduced toxicity, lower required doses with equal or improved effectiveness, and diminished development of drug resistance (Foucquier and Guedj, 2015). While single lipid-targeted medications can impede the growth and metastasis of cancer cells by inhibiting specific lipid-related pathways, they often fall short in completely eradicating cancer cells. In contrast, combination therapy exploits the synergistic interactions between different drugs, working in tandem to achieve the ultimate objective of eliminating cancer cells. By simultaneously targeting multiple pathways or molecular targets involved in lipid metabolism, combination therapy exhibits a more comprehensive and potent anticancer effect. Numerous pharmacological inhibitors have been developed to target various lipid-metabolizing enzymes, and when combined with conventional therapies, they have demonstrated significant therapeutic efficacy (refer to Table 1 for examples). The rationale behind combination therapy lies in the complementary mechanisms of action and additive or synergistic effects that arise from targeting multiple points within the lipid metabolic pathways. Overall, combination therapy holds great promise as a powerful strategy to combat diseases, particularly in the context of lipid metabolism. The judicious selection and integration of lipid-targeted drugs in combination regimens can lead to improved treatment outcomes and offer new avenues for overcoming drug resistance. Continued exploration of optimal drug combinations and their mechanisms of action will undoubtedly contribute to advancements in therapeutic approaches for various disorders.

**TABLE 1:** Lipid metabolism-related combination therapy alleviates tumor resistance.

Pathway/Enzyme	Lipid targeted drug	Drug combination	Vivo or vitro models	Effects
**FAS**	Orlistat	Trastuzumab	Chemotherapy-resistant ovarian cancer cells	Apoptosis increased significantly Menendez et al. (2006)
		Taxanes	Prostate resistant cell lines	Decreases viability increases apoptosis Souchek et al. (2017)
**SCD1**	A939572	Gefitinib	Lung cell lines	Reduces tumor progression and inhibits cancer cells She et al. (2019)
		Temsirolimus	Clear renal cell carcinoma cell lines	Decreases tumor cell proliferation and induction of apoptosis von Roemeling et al. (2013)
	SSI-4	Sorafenib	Sorafenib-resistant hepatocellular carcinoma cell lines	Increased sensitivity to sorafenib Ma et al. (2017)
	SSI-4	5-fluorouracil cisplatin	Gastric cells	Increased sensitivity to5-fluorouracil and cisplatin Wong et al. (2023)
	g-PPT	Gefitinib	TKI-resistant non-small cell lung cancer cell lines	Reverses resistance Huang et al. (2019)
**LPCAT2**	LPCAT2 or LD biogenesis inhibitor	Oxaliplatin	Colorectal cancer	Relieve drug resistance Cotte et al. (2018)
		5-fluorouracil		
**Glycolytic ferment**	PFKFB3	Carboplatin paclitaxel	Cervixcancer	Increased sensitivity to carboplatin and paclitaxel Mondal et al. (2019)
**HMG-CoA reductase**	Statins	Cytarabine, Daunorubicin, Doxorubicin, etc.	Colon cancer	Growth inhibition increased apoptosis Ding et al. (2017), Stirewalt et al. (2003)
			Breast cancer	
**FABP**	BMS309403	Carboplatin	Carboplatin-resistant ovarian cancer cell lines	Increased sensitivity to carboplatin Mukherjee et al. (2020)
**CPT1**	Etomoxir	Ara-C	Drug-resistant leukemia cells	Enhanced the cytotoxicity of Ara-C87 Salunkhe et al. (2020)

In the context of drug-resistant ovarian cancer, the combination of the FAS inhibitor orlistat and the specific Her-2 inhibitor trastuzumab has shown remarkable synergistic effects. *In vitro* studies demonstrated a substantial increase in apoptosis among chemotherapy-resistant ovarian cancer cells upon treatment with this combination (Menendez et al., 2006). Similarly, in prostate-resistant cell lines, the combination of orlistat with paclitaxel analogs exhibited reduced cell viability and increased apoptotic activity (Souchek et al., 2017). These findings highlight the potential of combination therapy in overcoming drug resistance and enhancing treatment outcomes in specific types of cancer. By concurrently targeting distinct molecular pathways or cellular processes, such as FAS inhibition and Her-2 blockade, orlistat and trastuzumab acted synergistically to induce apoptosis and impede the survival of drug-resistant ovarian cancer cells. Likewise, the combination of orlistat with paclitaxel analogs demonstrated enhanced efficacy in prostate-resistant cell lines, further underscoring the benefits of combining drugs with different mechanisms of action. The use of combination therapies holds great promise in addressing the challenges posed by drug resistance in cancer treatment. By exploiting synergistic interactions between drugs, these regimens offer the potential for improved therapeutic outcomes, reduced drug resistance, and enhanced patient responses. Continued research into optimal drug combinations and their underlying mechanisms will undoubtedly advance our understanding and implementation of combination therapies in combating drug-resistant cancers.

The SCD1 enzyme activity inhibitor A939572 has shown promising results in combination with gefitinib, a targeted therapy for lung cancer. When used together, A939572 and gefitinib significantly impeded tumor progression, inhibited cancer cell growth, and exhibited favorable outcomes in an *in vivo* xenograft model (She et al., 2019). Similarly, in renal clear carcinoma cells and transplanted tumors, the combination of A939572 with tesirolimus demonstrated inhibitory effects on tumor cell proliferation and facilitated apoptosis both *in vitro* and *in vivo* (von Roemeling et al., 2013). Another SCD1 inhibitor, SSI-4, has also displayed potential in overcoming drug resistance in different cancer types. In hepatocellular carcinoma cells resistant to sorafenib, the combination of SSI-4 with sorafenib restored sensitivity to sorafenib and exhibited significant therapeutic benefits (Ma et al., 2017). Moreover, SSI-4 improved the sensitivity of gastric cancer-resistant cells to treatment with 5-fluorouracil and cisplatin (Wong et al., 2023). g-PPT, another SCD1 inhibitor, has demonstrated efficacy in reducing the synthesis of polyunsaturated fatty acids, inhibiting triglyceride (TG) synthesis, and preventing lipid droplet accumulation in cancer cells. In TKI-resistant non-small-cell lung cancer cells, the combination of g-PPT with gefitinib effectively countered drug resistance, promoting apoptosis and enhancing the therapeutic response (Huang et al., 2019). These findings highlight the potential of SCD1 inhibitors in combination with existing therapies for overcoming drug resistance and improving treatment outcomes in various cancer types. By targeting lipid metabolism pathways and modulating cellular processes, such combinations offer a promising approach to tackle drug resistance and enhance the efficacy of existing treatments. Continued research and clinical investigations are warranted to validate and further explore the potential benefits of these combination regimens in cancer therapy.

In colorectal cancer, increased LPCAT2-mediated lipid droplet (LD) production has been linked to resistance against oxaliplatin and 5-fluorouracil (Cotte et al., 2018). In subsequent *in vivo* experiments using a colon cancer mouse model, the administration of LPCAT2 or LD biogenesis inhibitors resulted in tumor regression and increased survival, indicating the significant improvement of LPCAT2-mediated drug resistance. Similarly, in mice with ovarian and cervical cancers that were insensitive to carboplatin and paclitaxel, the use of the glycolytic enzyme inhibitor PFKFB3 showed promising results. By indirectly blocking LD biogenesis and lipid autophagy, PFKFB3 alleviated resistance to carboplatin and paclitaxel, thereby enhancing their effectiveness (Mondal et al., 2019). Furthermore, studies conducted on rat H35 and human HepG2 cells, known to be resistant to various antitumor agents, revealed the potential of lovastatin in reversing chemotherapeutic resistance (Le Bras et al., 2006). Lovastatin, through its inhibition of HMGCR, a key enzyme in cholesterol synthesis, effectively prevented the accumulation of cholesterol in the mitochondrial membrane. This inhibition of cholesterol synthesis in mitochondria by lovastatin led to the restoration of sensitivity to chemotherapeutic agents in H35 and HepG2 cells. These findings underscore the significance of targeting lipid metabolism pathways and LD biogenesis to combat drug resistance in cancer cells. Inhibition of LPCAT2-mediated LD production or modulation of glycolytic enzymes and cholesterol synthesis holds promise for overcoming resistance to specific chemotherapeutic agents and improving treatment outcomes. Further research is needed to explore the full potential of these approaches and their applicability in clinical settings.

Moreover, statins, inhibitors of the 3-hydroxy-3-methylglutaryl coenzyme A (HMG-CoA) reductase enzyme, have demonstrated the ability to inhibit cancer cell growth and induce apoptosis in various types of tumor cell lines. When used in combination with drugs like cytarabine, erythromycin, and doxorubicin, statins have shown promising results in xenograft models of colon cancer, breast cancer, and other malignancies (Stirewalt et al., 2003; Ding et al., 2017). These findings suggest the potential of statins as adjunctive therapy to enhance the efficacy of existing chemotherapeutic agents. Another noteworthy inhibitor, BMS309403, which targets fatty acid-binding proteins (FABPs), has shown promise in overcoming resistance to carboplatin in ovarian cancer. When combined with carboplatin, BMS309403 significantly increased the sensitivity of carboplatin-resistant cells, offering a potential strategy to overcome resistance in this context (Mukherjee et al., 2020). These findings highlight the potential of combining statins or FABP inhibitors with conventional chemotherapeutic agents to improve treatment outcomes and overcome drug resistance in various cancer types. Further research and clinical studies are warranted to explore the full therapeutic potential and safety profile of these combinations.

Another interesting study focused on leukemia cells that had developed resistance to the chemotherapeutic agent arabinofuranosylcytosine (Ara-C). In these resistant cells, it was found that Ara-C preferentially drove the tricarboxylic acid (TCA) cycle through fatty acids (FAs), relying less on glucose metabolism. This metabolic adaptation led to enhanced oxidative phosphorylation in the mitochondria (OXPHOS), which contributed to the cells’ drug resistance (Farge et al., 2017). Interestingly, the entry of long-chain fatty acids (LCFAs) into the mitochondria requires the involvement of carnitine palmitoyltransferase (CPT), which consists of two isoforms, CPT1 and CPT2 (Wang et al., 2020). In subsequent experiments, researchers discovered that blocking CPT1 using etomoxir, a specific inhibitor, disrupted the OXPHOS status and significantly potentiated the cytotoxic effect of Ara-C on drug-resistant leukemia cells (Salunkhe et al., 2020). This finding suggests that targeting fatty acid metabolism through CPT1 inhibition could be a promising approach to sensitize resistant leukemia cells to Ara-C treatment. These findings shed light on the metabolic reprogramming occurring in drug-resistant leukemia cells and highlight the potential of targeting specific metabolic pathways, such as fatty acid metabolism, to overcome drug resistance and improve therapeutic outcomes. Further investigations are needed to validate these findings and explore the clinical implications of modulating fatty acid metabolism in the context of leukemia treatment.

Lipid metabolism plays a crucial role in the development of resistance to anti-angiogenic drugs (AAD). Researchers have identified that combining anti-angiogenic therapy with lipid metabolism inhibitors could potentially overcome the emergence of AAD resistance. Preclinical studies have demonstrated that tumors with identical genetic backgrounds but implanted in different locations exhibit varying responses to AAD treatment (Iwamoto et al., 2018). For instance, hepatocellular carcinoma (HCC) growing in a steatotic (fatty) liver becomes resistant to anti-angiogenic therapy, while HCC growing in a non-steatotic liver remains sensitive. These findings highlight the influence of the adipose tissue environment on AAD resistance. Tumors growing in a fatty environment are typically more hypoxic compared to those developing in non-adipose tissues. Hypoxia induced by AAD leads to three significant alterations in lipid metabolism. Firstly, it promotes lipolysis in adipocytes, leading to the release of metabolites such as glycerol and free fatty acids (FFAs) (Nieman et al., 2011). Secondly, cancer cells respond to hypoxia by upregulating the expression of fatty acid translocase (CD36), facilitating increased uptake of FFAs by the tumor cells (Choi et al., 2018). Lastly, cancer cells undergo metabolic reprogramming, activating the *β*-oxidation pathway to generate energy from FFAs, thereby promoting tumor growth and metastasis (Park et al., 2016). Building upon these findings, combining AAD with inhibitors targeting lipid metabolism holds promise in alleviating and overcoming the development of AAD resistance. By targeting the metabolic adaptations occurring in the tumor microenvironment, this combination approach has the potential to enhance the effectiveness of anti-angiogenic therapy and improve treatment outcomes. However, further research and clinical investigations are necessary to validate these findings and determine the optimal strategies for combining AAD with lipid metabolism inhibitors in the clinical setting.

## 7 Summary and discussion

Malignant tumors pose a significant global health threat, and their incidence continues to rise each year (Ferlay et al., 2018). While medical advancements, have greatly improved the overall survival rates (Weiss et al., 2022), drug resistance complicates treatment strategies. Thus, overcoming drug resistance has become a critical issue in anticancer therapy. Recent studies have highlighted the role of lipid metabolism in influencing drug resistance (Kramer et al., 2015; Wu et al., 2015; Cotte et al., 2018). It has been shown that lipid-related processes can impact drug efficacy by affecting drug diffusion, altering membrane permeability, influencing mitochondrial function, and modulating the activity of ABC transporter proteins (Hegedüs et al., 2015). Upregulation of LPCAT2 and SCD1(Schlaepfer et al., 2012; Cotte et al., 2018), enhanced lipid droplet formation (Hultsch et al., 2018), PL precursor LysoPL and LPA-1 in phospholipid metabolism (Kramer et al., 2015) are all associated with drug resistance. Cholesterol metabolism affects drug penetration, absorption, and drug resistance by affecting mitochondrial cholesterol level (Le Bras et al., 2006), ABC transporter activity (Waghray and Zhang, 2018) and cell membrane cholesterol content (Subramanian et al., 2016). This review details the changes of lipid metabolism in drug resistance and how lipid metabolism affects drug resistance.

Further studies have shown that while single lipid-targeting drugs can hinder cancer cell growth and metastasis by inhibiting specific lipid-related pathways, they often fail to completely eradicate cancer cells (Han et al., 2017). In contrast, combination therapy utilizes synergistic interactions between different drugs that work together to reach the ultimate goal of eliminating cancer cells. Combination therapy shows a more comprehensive and effective anti-cancer effect by simultaneously targeting multiple pathways or molecular targets of lipid metabolism (Foucquier and Guedj, 2015). This review summarizes the progress of drug design targeting lipid metabolism in improving drug resistance.

The advantages of drug combination and our understanding of lipid metabolism suggest that we can continue to explore the synergies between targeted drugs of lipid metabolism and traditional anticancer drugs, so as to innovate new therapeutic approaches to improve the efficacy of anticancer drugs and mitigate the emergence of drug resistance for the benefit of patients. Therefore, further research in this area is essential to uncover the complexity of lipid metabolism during tumor resistance and to optimize the implementation of combination therapy strategies.
